# Autophagy-associated circRNA circATG7 facilitates autophagy and promotes pancreatic cancer progression

**DOI:** 10.1038/s41419-022-04677-0

**Published:** 2022-03-14

**Authors:** Zhiwei He, Kun Cai, Zhirui Zeng, Shan Lei, Wenpeng Cao, Xiaowu Li

**Affiliations:** 1 Department of Hepatobiliary Surgery, Shenzhen Key Laboratory, Shenzhen University General Hospital, Shenzhen, Guangdong 518055 China; 2 Guangdong Key Laboratory for Biomedical Measurements and Ultrasound Imaging, School of Biomedical Engineering, Shenzhen University Health Science Center, Shenzhen, 518060 China; 3 Guangdong Provincial Key Laboratory of Regional Immunity and Diseases & Carson International Cancer Center, Shenzhen University, Shenzhen, Guangdong 518055 China; 4 Shenzhen University Clinical Medical Academy Center, Shenzhen University, Shenzhen, Guangdong 518055 China; 5 Guizhou Medical University, Guiyang, China; 6 Department of Hepatic-Biliary-Pancreatic Surgery, The Affiliated Hospital of Guizhou Medical University, Guiyang, 550000 Guizhou China; 7 School of Basic Medicine, Guizhou Medical University, Guiyang, 550025 Guizhou China

**Keywords:** Pancreatic cancer, Pancreatic cancer

## Abstract

Dysregulation of autophagy and circular RNAs (circRNAs) are involved in the pancreatic cancer (PC) progression. However, the regulatory network between circRNAs, autophagy, and PC progression remains unknown. Herein, we demonstrated that autophagy-associated circRNA circ-autophagy related 7 (circATG7) was elevated in PC tissues compared to adjacent tissues, and in PC cells treated with EBSS and hypoxia. circATG7 expression was positively associated with tumor diameter and lymph node invasion in patients with PC. circATG7 overexpression promoted PC cell proliferation, mobility, and autophagy in vitro, while circATG7 knockdown induced the opposite effects. ATG7 inhibition attenuated the effects of circATG7 on the biological functions of PC cells. CircATG7 is located in the cell cytoplasm and nucleus. Cytoplasmic circATG7 sponged miR-766-5p and decreased its expression, and increased the expression of ATG7, a target gene of miR-766-5p. Nuclear circATG7 acted as a scaffold to increase the interaction between the human antigen R protein and ATG7 mRNA and enhanced ATG mRNA stability. Furthermore, we demonstrated that circATG7 regulates PC cell proliferation and metastasis in vivo via ATG7-dependent autophagy. In conclusion, our results demonstrated that circATG7 accelerates PC progression via miR-766-5p/ATG7 and that HUR/ATG7 depends on autophagic flux. Thus, circATG7 may be a potential therapeutic target for PC.

## Introduction

Pancreatic cancer (PC) is responsible for the fourth highest number of tumor-related deaths worldwide [1]. Although therapeutic options for patients with PC have improved, the 5-year survival rate for patients with PC is still <5% [2, 3]. The poor prognosis for patients with PC is caused by the high proliferation and early invasion of cancer cells. Therefore, there is an urgent need to elucidate the molecular mechanisms involved in the progression of PC [4].

Autophagy is a stress response in cells, which can protect them from damage [5]. Abnormal autophagy has been observed in multiple diseases, including cardiac-cerebral vascular diseases [6], neurodegenerative diseases [7], and cancers. The role of autophagy in PC is complex, moderate autophagy promotes the proliferation and invasion of PC cells, as well as drug resistance through the breakdown of damaged organs, producing substances such as amino acids; however, excessive autophagy leads to PC cell apoptosis due to organelle injury and the degradation of pro-oncogenes [8]. Autophagy-related 7 (ATG7) is a critical protein involved in autophagy, which activates ATG12 for conjugation with ATG5 as well as the ATG8 family proteins for conjugation with phosphatidylethanolamine [9]. Inhibiting autophagy via the knockdown of ATG7 has been shown to suppress the proliferation and metastasis of PC cells [10]. However, the regulatory mechanisms of ATG7 in PC remain largely unknown.

Circular RNAs (circRNAs), a class of novel noncoding RNAs, were discovered in 1976. CircRNAs derive from non-canonical splices of pre-mRNAs, and form a covalently closed loop structure lacking 5′ caps and 3′ tails [11]. CircRNAs present higher stability and resistance to exonuclease compared to the parental linear RNAs [12]. Previous studies have demonstrated that circRNAs regulate the biological functions of cancer cells by sponging microRNAs (miRNAs) [13] and maintaining the stability of parental genes [14]. Interestingly, some circRNAs promote the progression of tumors by directly translating proteins [15]. Circ-argonaute RISC catalytic component 2 was shown to accelerate tumor progression by physically interacting with human antigen R (HUR) protein, facilitating its activation and decreasing the degradation of target genes [16]. Circ-nuclear receptor-interacting protein 1 was found to sponge with miR-149-5p, activate the AKT/mTOR pathway, and promote the progression of gastric carcinoma [17]. Circ-F-box and WD repeat domain-containing 7 encodes a novel 21 kDa protein, which promotes the progression of glioma [15]. Several circRNAs were also found to regulate autophagy, including circ-chromodomain Y like [18] and circ-mucin 16 [19]. In pancreatic cancer, circ-nuclear factor IB1 [20] and circ-low density lipoprotein receptor class A domain containing 3 [21] can act as biomarkers to predict patient prognosis. However, the relationship between circRNAs, autophagy, and PC progression remains limited.

In the current study, we demonstrated that expression of an autophagy-associated circRNA circ-autophagy-related 7 (circATG7), derived from the exon region of the ATG7 gene, was elevated in PC tissues and associated with a lower survival rate for patients with PC. CircATG7 promotes the proliferation and motility of PC cells by regulating miR-766-5p/ATG7 and HUR/ATG7 axis-mediated autophagy. Thus, autophagy-associated circATG7 may be an effective therapeutic target for patients with PC.

## Materials and methods

### Sample collection

92 cases tumor tissue and adjacent normal tissue were collected from patients with PC who underwent surgery at the Department of Hepatobiliary Surgery, Affiliated Hospital of Guizhou Medical University (Guiyang, China), and used for reverse transcription-polymerase chain reaction (RT-qPCR) and in situ hybridization (ISH) analyses. Sample collection and usage were approved by the Ethics Committee of Guizhou Medical University in accordance with the Declaration of Helsinki, and written informed consent was obtained from all enrolled patients.

### Cell culture

The pancreatic epithelial cell lines HPDE and PC (AsPc-1, BXPC-3, Capan-2, CFPAC-1, PANC-1, MIA-PaCa2, and SW1990) were obtained from the American Type Culture Collection. HPDE, AsPc-1, and BXPC-3 cells were maintained in RPMI-1640 medium (Gibco) containing 10% fetal bovine serum (FBS; Gibco), while Capan-2, CFPAC-1, PANC-1, MIA-PaCa2, and SW1990 were cultured in DMEM containing 10% FBS. All cells were cultured in a humidified environment with 5% CO_2_ at 37 °C. All the cell lines had been authenticated by STR profiling and tested for mycoplasma-free.

### Bioinformatics analysis

To analyze the expression of circATG7 in PC, the circRNA expression profile GSE69362, contributed by Qu S et al. [22], was downloaded from the Gene Expression Omnibus database (https://www.ncbi.nlm.nih.gov/gds). This contained circRNA expression data from six adjacent normal pancreatic and PC tissues. After normalization, a paired t-test was used to analyze the differences in circATG7 expression between adjacent normal pancreatic tissues and PC tissues. Differences were considered statistically significant at *p* < 0.05.

### RT-qPCR

Total RNA was extracted from PC cells and tissues using TRIzol reagent (Takara). To determine mRNA and circRNA expression, cDNA was reverse transcribed using the iSCRIPT cDNA synthesis Kit (Roche® Life Science), while the miRNA First Strand cDNA Synthesis Kit (Sangon) was used to reverse transcribe miRNA as cDNA. Amplification was performed on a CFX96 Touch Real-Time Fluorescence Quantitative PCR Instrument (Bio-Rad Laboratories) using TB Green® Fast qPCR Mix (Takara). GAPDH (mRNA and circRNA) and U6 (miRNA) were used as loading controls. The results were analyzed using the 2 ^-ΔΔCt^ method. The primer sequences used in the present study were as follows:

circATG7 Forward primer (5′ to 3′): CTCCTCTTGACATTTGCAGAGTG,

circATG7 Reverse primer (5′ to 3′): GCAGTCTTGAAAGACTCGAGTGTG;

miR-766-5p Forward primer (5′ to 3′): TCGAGTACTTGAGATGGAGTTTT,

miR-766-5p Reverse primer (5′ to 3′): GGCCGCGTTGCAGTGAGCCGAG;

ATG7 Forward primer (5′ to 3′): CAGTTTGCCCCTTTTAGTAGTGC,

ATG7 Reverse primer (5′ to 3′): CCAGCCGATACTCGTTCAGC;

U6 Forward primer (5′ to 3′): CTCGCTTCGGCAGCACA,

U6 Reverse primer (5′ to 3′): AACGCTTCACGAATTTGCGT;

GAPDH Forward primer (5′ to 3′): CTCCAAAATCAAGTGGGGCG,

GAPDH Reverse primer (5′ to 3′): TGGTTCACACCCATGACGAA.

### In situ hybridization

The expression of CircATG7 in PC and adjacent normal tissues was analyzed using biotin-labeled circATG7 probes (Guangzhou RiboBio Co., Ltd.). Paraffinized PC tissues were deparaffinized with different concentrations of xylene and ethanol. The PC tissues were then incubated with circATG7 probes overnight at 40 °C. Digoxin substrate was used to visualize circATG7 signals, and cell nuclei were stained with hematoxylin.

### RNase R digestion

Total RNA (4 μg) was isolated from PC cells using TRIzol reagent, and the samples were divided into experimental and control groups. The experimental groups were treated with RNase R (4 U/μg) for 30 min at 37 °C, whereas the control group was treated with solvent. The products were then analyzed using RT-qPCR as previously described.

### Nuclear-cytoplasmic RNA isolation

Cytoplasmic and nuclear RNAs were isolated using the PARIS kit (Life Technologies) according to the manufacturer’s instructions. The amount of circATG7 was detected by RT-qPCR, while GADPH and U6 acted as reference genes for cytoplasmic and nuclear RNA, respectively.

### Fluorescence in situ hybridization (FISH)

Cy3-labeled circATG7 probes (synthesized by Guangzhou RiboBio Co., Ltd.) were used to determine the expression and subcellular localization of circATG7 in PC cells. PC cells were attached to coverslips, and 4% paraformaldehyde (Solarbio Life Sciences) and protease K (Invitrogen) were used to fix and digest the cells, respectively. The cells were then dehydrated through an alcohol gradient (70, 85, and 100%). CircATG7 probes were diluted in hybridization solution. Following degeneration, the probes were incubated overnight in the dark at 40 °C. Following incubation with formamide, NP-40, and DAPI, probe signals were detected using a laser scanning confocal microscope (Nikon).

### Cell transfection and lentiviral infection

CircATG7-overexpressing lentivirus, short hairpin RNAs targeting circATG7, and their corresponding controls were obtained from Genechem (Shanghai, China). The MiR-766-5p mimic and negative control mimic were purchased from Genechem. Small interfering RNAs (siRNAs) for ATG7 and HUR were obtained from Guangzhou RiboBio Co., Ltd. Transient transfection of mimics and siRNAs was performed using Lipo2000 (Invitrogen), according to the manufacturer’s instructions. Following lentiviral infection for 48 h, PC cells were cultured with puromycin (1 μM, Invitrogen) for 2 weeks to construct stable-expressing cell lines.

### Cell proliferation assays

The proliferation of PC cells was evaluated using the Cell Count Kit-8 (CCK-8) and 5-ethynyl-2′-deoxyuridine (EDU) assays. For the CCK-8 assay, PC cells were seeded into a 96-well plate at a density of 3 × 10^3^, and six duplicate wells were used for each experimental group. After culturing for 0 h (cell adherence), 24, 48, and 72 h at 37 °C, 100 μL medium containing 10 μL CCK-8 reagent (GlpBio Technology) was added into each well, and the absorbance value (OD) of each well was detected at 450 nm using an iMark microplate reader (Bio-Rad Laboratories) followed by 2 h of culture. The EDU assay was performed using the EdU Apollo 562 kit (Guangzhou Ribobio Co., Ltd.), and EDU-positive cells were visualized using an IX51-FL fluorescence microscope (Olympus-Lifescience).

### Colony formation assay

A total of 800 PC cells were seeded into a six-well plate and cultured for 14 days at 37 °C. The medium was then disposed, and the cell colonies were fixed with 4% paraformaldehyde. Cells were washed with PBS and stained with 0.5% crystal violet solution (Solarbio Life Sciences) for 30 min, and then the number of cell colonies was counted.

### Transwell assay

The migration and invasion ability of PC cells were determined using 8 μm migration Transwell chambers (Corning) and Transwell chambers pre-coated with Matrigel (Becton, Dickinson and Company). Briefly, 5 × 10^4^ PC cells in 200 μL serum-free DMEM were seeded in the upper chambers. DMEM (700 μL) containing 10% FBS was placed in the lower chambers as an inductive substance. After 24 h of culture, the upper chamber was fixed with 4% paraformaldehyde for 20 min and stained with 1% crystal violet. After removing non-migrated and non-invasive cells, the migrated and invasive cells were photographed using a BX optical microscope (Olympus Corporation).

### Immunofluorescence and confocal microscopy

The sections were fixed and permeabilized with 0.3% Triton (Solarbio Life Sciences) for 10 min. After blocking non-specific binding using 5% BSA, sections were incubated with LC3 (1:100, Cat No. 14600-1-AP, Proteintech Group, Inc), E-cadherin (1:100; Cat No. 20874-1-AP, Proteintech Group, Inc.), and vimentin (1:100; Catalog number: 10366-1-AP, Proteintech Group, Inc.) antibodies overnight at 4 °C. After washing with PBS three times, sections were incubated with FITC-labeled goat anti-rabbit secondary antibody (1:50; Catalog number: SA00003-2, Proteintech Group, Inc.). The cell nuclei were stained with DAPI, and the expression of target proteins was visualized using a fluorescence microscope. For confocal microscopy, after transfecting RFP-GFP-LC3 adenovirus for 48 h, PC cells were fixed, and autophagosome dots were photographed and counted using a laser scanning confocal microscope.

### Transmission electron microscopy (TEM)

PC cells were fixed with 2.5% glutaraldehyde and 1% osmium tetroxide at 4 °C and immersed in spur resin after dehydration. The cells were then stained with 4% uranyl acetate and lead citrate. Finally, images were captured using a TEM (Hitachi, Ltd).

### Western blot

Total protein was extracted from cells using RIPA reagent (Boster Biological Technology Co., Ltd.) containing 1% PMSF (Boster Biological Technology Co., Ltd.). The BCA method was used to determine the concentration of protein in each sample. Protein samples were separated by SDS-PAGE and transferred to polyvinylidene difluoride membranes. The membranes were blocked with 5% BSA, and then incubated with ATG7 (1:500; Cat No. 10088-2-AP, Proteintech Group), ATG5-12 (1:500; Cat no. 12994, Cell Signaling Technology, Inc.), P62 (1:1000; Cat No. 18420-1-AP, Proteintech Group), LC3 (1:1000), HUR (1:500; Cat No. 12582, Cell Signaling Technology, Inc.), and GAPDH (1:500; Cat No.10494-1-AP, Proteintech Group) primary antibodies overnight at 4 °C. After washing with TBST three times, horseradish peroxidase-labeled goat anti-rabbit secondary antibody (Boster Biological Technology Co. Ltd.) was added for 2 h. Bands were then visualized using ECL reagent (Boster Biological Technology Co. Ltd.) with a chemiluminescence imaging system (Bio-Rad). All the full and uncropped western blots are uploaded as ‘Supplemental Material’.

### Luciferase reporter assay

PC cells (5 × 10^5^) were seeded into six-well plates. Then, the miR-766-5p mimics and corresponding negative control mimics were co-transfected in the relevant cells. The cells were then lysed, and firefly and Renilla luciferase activities were determined. Finally, firefly luciferase was used to normalize the relative luciferase activity.

### RNA pull-down assay and RNA immunoprecipitation

PC cells were lysed and incubated with biotin-labeled circATG7 probes and normal probes overnight at 4 °C. The pull-down products were isolated using an RNeasy Mini Kit (Qiagen, USA). The binding protein of circATG7 was analyzed by western blotting. For RNA immunoprecipitation, cells were cultured with biotin-labeled miR-766-5p probes and normal probes and lysed in complete RNA immunoprecipitation lysis buffer. Then, magnetic beads conjugated with anti-Argonaute 2 (AGO2) were added for enrichment. Proteinase K was used to remove proteins, and TRIzol reagent was used to collect RNAs. The purified RNA was subjected to qRT-PCR analysis.

### In vivo experiments

All protocols for animal experiments were approved by the Guizhou Medical University (Guiyang, China). Five nude mice were randomly selected for each group.PC cells were resuspended in PBS and the density was adjusted to 1 × 10^7^/mL. To evaluate proliferation in vivo, 200 μL cell suspension was injected into the right flank of mice (*n* = 5/group). The tumor volume was then measured every 5 days. Mice were euthanized on day 30, and the tumor tissues were extracted, weighing, and used for immunohistochemistry analysis. To evaluate metastasis in vivo, 100 μL cell suspension was injected into the caudal vein of mice. The mice were euthanized 8 weeks after injection, and metastatic foci in the lung were detected by HE staining.

### Immunohistochemistry

Tumor sections were dewaxed in xylene and dehydrated through a gradient of alcohol. Antigen retrieval was performed using a citrate buffer (pH 6.0) in a microwave. Then, non-specific binding was blocked using 3% H_2_O_2_ and 5% BSA. Primary antibodies, including those against KI67 (1:200; Cat No. 27309-1-AP, Proteintech Group), PCNA (1:200; Cat No. 10205-2-AP, Proteintech Group), P62 (1:100), and LC3 (1:100), were added to the sections and incubated for 16 h at 4 °C. Following incubation with secondary antibodies and DAB, the cell nuclei were stained with hematoxylin. The results were evaluated blindly by two independent pathologists.

### Statistical analysis

The results were analyzed using SPSS 19.0 (IBM) and are presented as mean ± standard deviation (SD). Differences between groups were analyzed using the unpaired Student’s t-test. Survival was analyzed using Kaplan–Meier curves. The relationship between circATG7, miR-766-5p, and ATG7 expression was analyzed using Pearson correlation analysis. Statistical significance was set at *P* < 0.05.

## Results

### CircATG7 was identified as a candidate autophagy-associated circRNA in PC

**Fig. 1:**
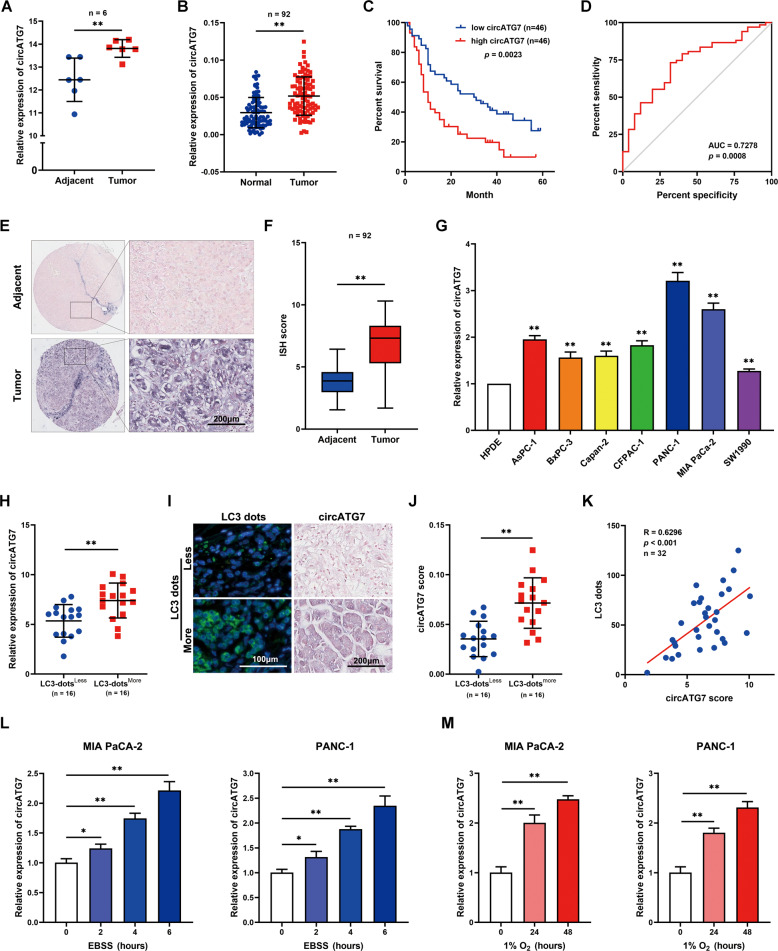
Autophagy-associated circRNA circATG7 was increased in PC tissues. **A** has_circ_0064288(circATG7) expression in PC tissues and adjacent tissues based on the expression profile of GSE69362. **B** RT-qPCR detected the expression of circATG7 in PC tissues and adjacent tissues. **C** Kaplan–Meier survival analysis showing the overall survival for patients with PC and high or low circATG7 expression. **D** Receiver operating character curve was obtained to analyze the diagnostic value of circATG7. **E**, **F** In situ hybridization was performed to determine the expression of circATG7 in PC tissues. **G** RT-qPCR was used to determine the expression of circATG7 in normal pancreatic epithelial (HPDE) and PC (AsPc-1, BXPC-3, Capan-2, CFPAC-1, PANC-1, MIA-PaCa2, and PANC-1) cell lines. **H** RT-qPCR was used to determine the expression of circATG7 in PC tissues with LC3 dots^less^ and LC3 dots^more^. **I**, **J** In situ hybridization was used to determine the expression of circATG7 in PC tissues with LC3 dots^less^ and LC3 dots^more^. **K** The relationship between circATG7 expression and LC3 dot number was determined by Pearson correlation analysis. **L** circATG7 expression in PC cells treated with EBSS. **M** circATG7 expression in PC cells cultured under hypoxic conditions. *P < 0.05; **P < 0.01.

To screen the autophagy-associated circRNA in PC, we developed a PRISMA flow to select the candidate circRNA (Supplementary Fig. 1A). Through analysis of the circRNA expression profile GSE69362, which contains data from six adjacent normal tissues and PC tissues, we found that a candidate circRNA, named has_circ_0064288(CircATG7), was increased in PC tissues compared to adjacent tissues (Fig. 1A). Similarly, we evaluated the expression of circRNA in 90 pairs of PC and adjacent normal tissues from our research group by RT-qPCR, and found that circATG7 expression was clearly increased in PC tissues (Fig. 1B). According to the median expression level of circATG7, samples from patients with PC were divided into high- and low-expression groups. Kaplan–Meier survival analysis revealed that high expression of circATG7 was associated with a lower overall survival rate (Fig. 1C). Receiver operator characteristic curve indicated that a distinguishable value of circATG7 could be used to estimate PC and adjacent normal tissues (Fig. 1D). Moreover, we found that circATG7 expression was positively associated with tumor diameter and lymph node invasion in patients with PC (Table 1). ISH and qRT-PCR assays showed that circATG7 expression was higher in PC tissues and cell lines compared to adjacent normal tissues (Fig. 1E, F) and normal pancreatic epithelium HPDE (Fig. 1G), respectively. The highest expression levels were found for PANC-1 and MIA-PaCa2.

**Table 1:** The relationship between circATG7 expression and PC clinical traits.

Clinicopathologic Feature		circATG7		*p-*value
		High expression	Low expression	
All cases		46	46	
Age				
≤50		9	5	0.2456
>50		37	41	
Gender				
male		28	24	0.4002
female		18	22	
Diameter of tumor				
≤2		25	36	***0.0153***
>2		21	10	
TNM stage				
I/II		40	43	0.2924
III/IV		6	3	
Lymphatic metastasis				
Negative		11	22	***0.0168***
Positive		35	24	
Distant metastasis				
Negative		41	44	0.2381
Positive		5	2	
Pathological grading				
I/II		32	28	0.3813
III/IV		14	18	

Bold entries indicate statistically significant *p*-values.

It is confirmed that autophagy plays a key role in the progression of PC, we evaluated the relationship between circATG7 and autophagy. PC tissues were divided into LC3 dots^Less^ and LC3 dots^More^ groups according to the median number of LC3 dots (Supplementary Fig. 1B). Meanwhile, the Kaplan–Meier survival analysis suggested that patients with more LC3 dots in the tumor tissues have a worse prognosis (Supplementary Fig. 1C). The results showed that circATG7 expression was increased in the PC samples with high LC3 dots (Fig. 1H–J), and was positively associated with the number of LC3 dots (Fig. 1K). Interestingly, circATG7 expression in PANC-1 and MIA-PaCa2 cells was significantly increased following treatment with EBSS and 1% O_2_ (Fig. 1L, M). Taken together, these results indicate that circATG7 may be a candidate autophagy-related circATG7.

### CircATG7 was derived from ATG7, and located in cytoplasm and nucleus

**Fig. 2:**
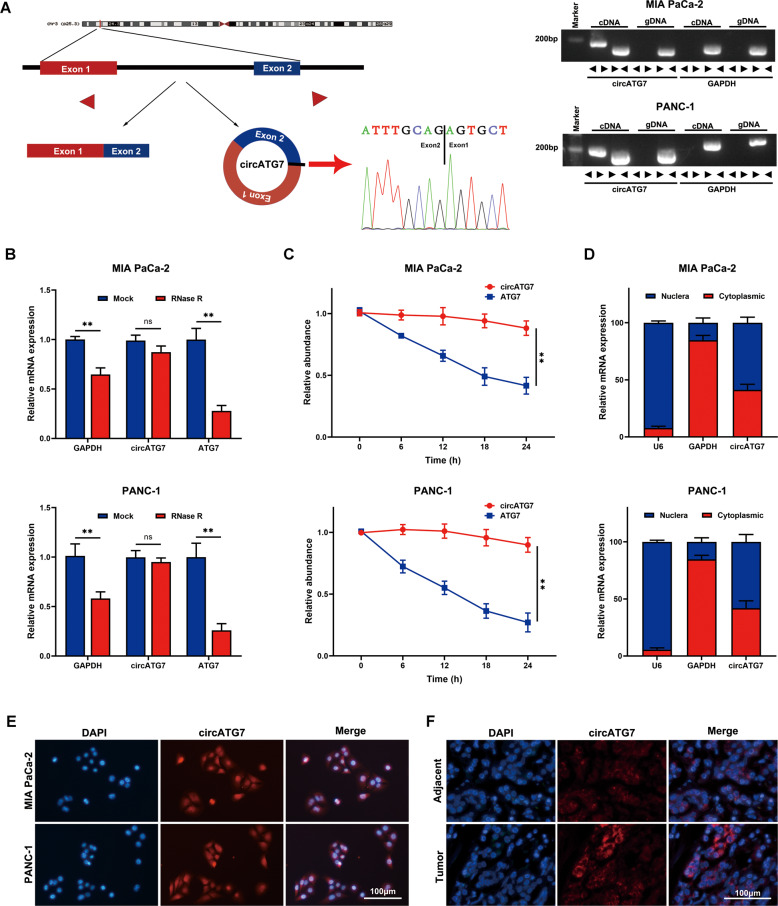
circATG7 is a conventional circRNA derived from ATG7 and is located in the cell nucleus and cytoplasm. **A** The circATG7 was back-spliced by ATG7 and verified by Sanger sequencing. **B** RT-qPCR was used to detect the expression of circATG7 and ATG7 in PC cells under RNase R treatment. **C** RT-qPCR was used to determine the expression of circATG7 and ATG7 in PC cells under amanitin treatment. **D, E** Nucleocytoplasmic fractionation and FISH revealed the subcellular localization of circATG7 in PC cells. **F** FISH revealed the subcellular localization of circATG7 in PC tissues and adjacent normal tissues. **P < 0.01.

Combining these results with circRNA annotation using the circBase database (http://www.circbase.org/), we found that circATG7 is located on chromosome 3 (chr3: 11348416–11350535), is 2119 bp long, and is generated by circularization of exons 1–2 of the host gene ATG7, The head-to-tail splicing of circATG7 was further verified by Sanger sequencing (Fig. 2A). We subjected cDNA and gDNA obtained from PANC-1 and MIA-PaCa2 cells to nucleic acid electrophoresis, the results showed that circATG7 was amplified by divergent primers in the cDNA, but not the extracted gDNA (Fig. 2B). As shown in previous studies, the lack of a 5′ cap and a 3′ polyadenylated tail provided circRNAs with more stability than linear mRNA [11]. Next, we treated PANC-1 and MIA-PaCa2 cells with RNase R, and evaluated degradation via RT-qPCR. The results showed that the expression of linear ATG7 mRNA was markedly reduced, while there was no significant change in the expression of circATG7 (Fig. 2C). Furthermore, we found that circATG7 decreased more slowly than linear ATG7 mRNA in PANC-1 and MIA-PaCa2 cells following treatment with amanitin (Fig. 2D). Nucleocytoplasmic fractionation (Fig. 2E) and FISH (Fig. 2F, G) further demonstrated that circATG7 was located in the cytoplasm and nucleus of PC cells and tissues.

### CircATG7 promoted the proliferation and motility of PC cells in vitro

**Fig. 3:**
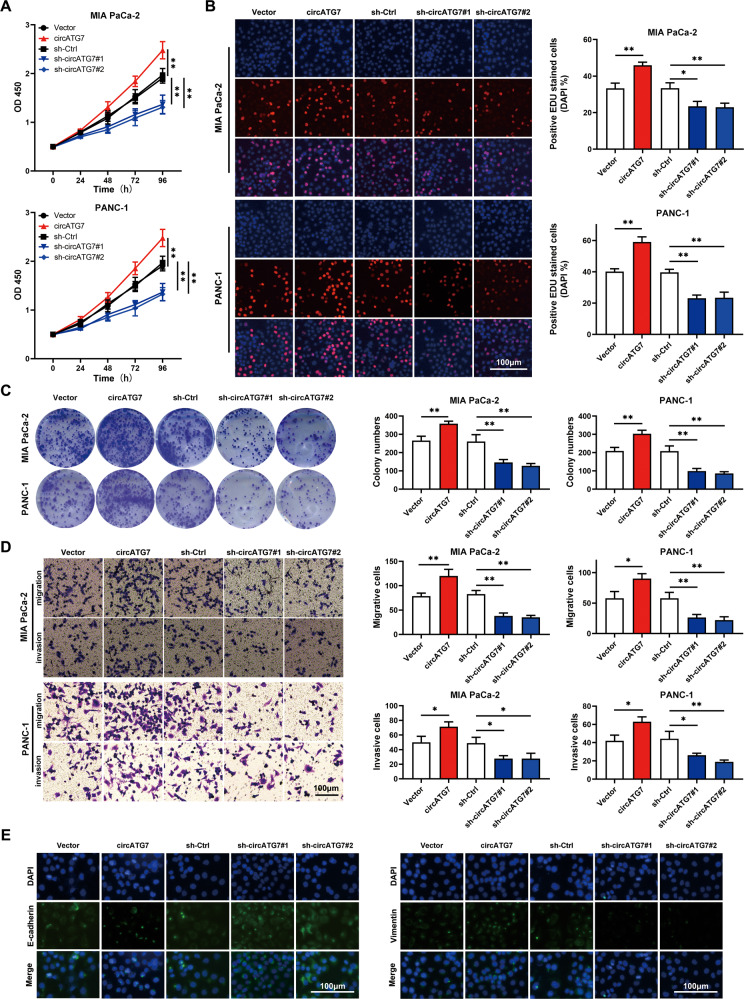
CircATG7 promotes the proliferation and mobility of PC cells in vitro. **A** CCK-8 was used to determine the proliferation of PC cells with circATG7 gain or loss. **B** EDU assay was used to determine the EDU positivity of PC tissues with circ-ATG7-overexpressing or knockdown. **C** Colony formation assay was used to detect the number of colonies in circATG7-overexpressing, circATG7-knockdown, and negative control groups. **D** Migration and invasion Transwell assays were used to determine the migration and invasion of PC cells with circ-ATG7 overexpression or knockdown. **E** E-cadherin and vimentin expression in circ-ATG7-overexpressing and knockdown group PC cells were determined using immunofluorescence staining. *P < 0.05; **P < 0.01.

To determine the role of circATG7 in PC cells, loss- and gain-of-function experiments were performed by transfecting circATG7 shRNAs and circATG7-overexpression lentivirus. The results of CCK-8 and EDU assays demonstrated that overexpression of circATG7 promoted the proliferation of PC cells, while inhibition of circATG7 decreased the proliferation of PC cells (Fig. 3A, B). The colony formation assay revealed that circATG7 overexpression increased the colony formation of PANC-1 and MIA-PaCa2 cells, while suppression of circATG7 exerted the opposite effect (Fig. 3C). Similarly, Transwell migration and invasion assays revealed that the number of migrated and invasive cells was increased in circATG7 overexpression groups and decreased in circATG7 knockdown groups compared to those in the control group (Fig. 3D). As shown previously, EMT is a key process in cancer cell metastasis; therefore, we then determined whether biomarkers of EMT were changed with the loss- and gain-of circATG7. Using immunofluorescence, reduced E-cadherin and increased vimentin levels were observed in circATG7-overexpressing PC cells, while circATG7 knockdown increased E-cadherin expression and resulted in the loss of vimentin (Fig. 3E).

### Inhibition of ATG7 reversed the stimulatory effects of circATG7 on the cell proliferation, motility, and autophagy of PC cells in vitro

**Fig. 4:**
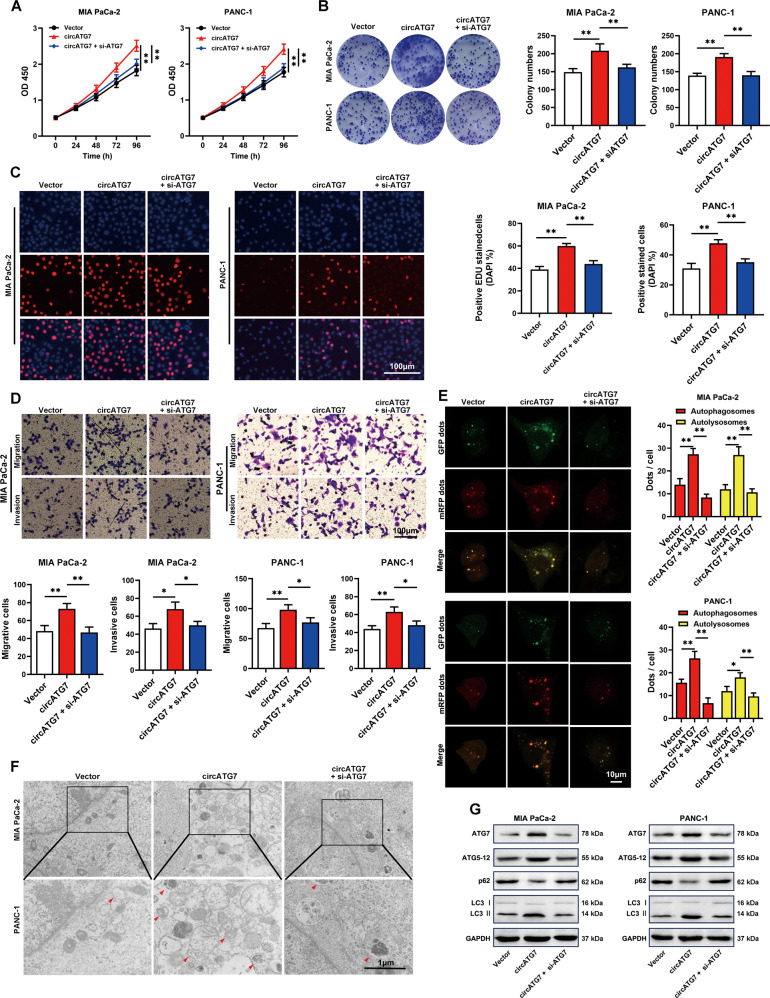
Knockdown of AGT7 reversed the promotive effects of circATG7 on PC cell proliferation, mobility, and autophagy. PC cells were treated with empty vector, circATG7-overexpressing lentivirus, and circATG7-overexpressing lentivirus plus ATG7 siRNA. **A** CCK-8 assay was used to determine the rate of PC cell proliferation in each group. **B** Colony formation assay was used to detect colony formation in each group. **C** EDU assay was performed to determine the rate of EDU positivity in each group. **D** Migration and invasion Transwell assays were used to determine the rates of migration and invasion in each group. **E**, **F** Confocal and transmission electron microscopy were used to assess autophagy in each group of PC cells. Red arrows represent autophagosomes. **G** Western blotting was used to detect biomarkers of autophagy in each group, including ATG7, ATG5-12, P62, and LC3. *P < 0.05; **P < 0.01.

circRNAs regulate biological functions via a parent-dependent mechanism [23]. Therefore, ATG7 siRNA was used, and we found that knockdown of ATG7 expression significantly decreased the accelerative effects of circATG7 on the proliferation of PC cells and colony formation (Fig. 4A–C). Similarly, through Transwell migration and invasion assays, we found that inhibition of ATG7 in PC cells with circATG7 overexpression decreased their ability for migration and invasion (Fig. 4D). As ATG7 is a key autophagy factor, we determined the level of autophagy in PC cells. By observing autophagy flux by confocal microscopy, circATG7 overexpression was found to increase the number of autophagosomes and autolysosomes in PC cells, while ATG7 suppression in circATG7-overexpressing cells blocked these effects (Fig. 4E). Similarly, TEM analysis revealed a significant increase in the number of autophagic vacuoles in circATG7-overexpressing PC cells compared with NC cells. Suppression of ATG7 was found to reverse the effects of circATG7 overexpression (Fig. 4F). Consistent with the results of autophagy flux observations and TEM, western blotting revealed that the expression of ATG7, ATG5-12, and LC3B II was increased in circATG7-overexpressing PC cells, while that of P62 was reduced. Inhibition of ATG7 in circATG7-overexpressing PC cells decreased the expression of ATG7, ATG5-12, and LC3B II and increased the expression of P62 (Fig. 4G).

### Cytoplasmic circATG7 increased the mRNA level of ATG7 via sponging miR-766-5p

**Fig. 5:**
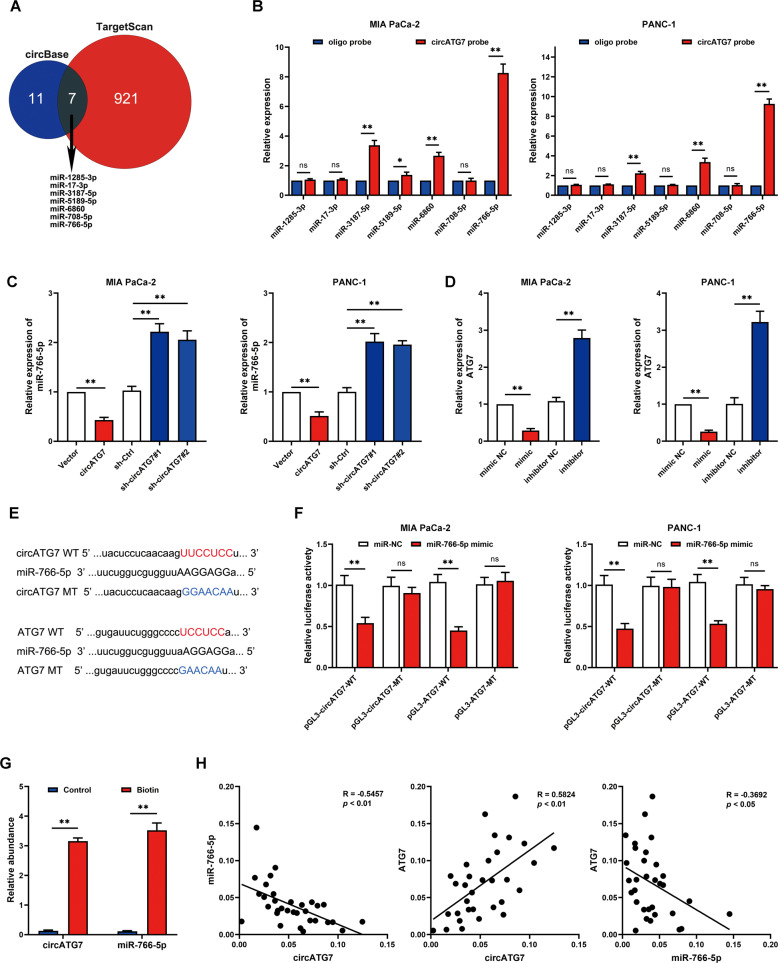
Cytoplasmic circATG7 increased the mRNA level of ATG7 via sponging miR-766-5p. **A** CircBase and TargetScan were used in combination to analyze potential miRNAs regulated by circATG7 that can bind with ATG7. **B** CircATG7 probes were used to analyze the enrichment of miR-1285-3p, miR-17-3p, miR-3187-5p, miR-5189-5p, miR-6860, miR-708-5p, and miR-766-5p. **C** miR-766-5p expression was detected by RT-qPCR in PC cells with circATG7 gain or loss. **D** ATG7 expression was detected by RT-qPCR in PC cells with miR-766-5p gain or loss. **E** Binding sites between circATG7, miR-766-5p, and ATG7 are shown. **F** Luciferase activity assay was used to detect the binding between circATG7, miR-766-5p, and ATG7. **G** circATG7 was significantly enriched in the miR-766-5p probes. **H** RT-qPCR was used to evaluate the relationship between circATG7, miR-766-5p, and ATG7 expression. *P < 0.05; **P < 0.01.

Cytoplasmic non-coding RNAs commonly regulate biological functions via a competing endogenous RNA mechanism [24]. As a certain proportion of circATG7 is located in the cytoplasm, we examined whether circATG7 regulated the expression of ATG7 by regulating miRNAs. Bioinformatics analysis in circBase (http://www.circbase.org/) predicted 18 miRNAs regulated by circATG7. Then, we predicted the miRNAs that had the potential to regulate ATG7 in TargetScan (http://www.targetscan.org/vert_72/), 928 miRNAs were identified. Therefore, intersection analysis identified seven miRNAs, including miR-1285-3p, miR-17-3p, miR-3187-5p, miR-5189-5p, miR-6860, miR-708-5p, and miR-766-5p, which are regulated by circATG7 and have the potential to regulate ATG7 (Fig. 5A). Among these, miR-766-5p was most enriched in circATG7probes in Mia-PaCa2 and PANC-1 (Fig. 5B). And we also found that miR-766-5p expression was downregulated and positive with the prognosis in the pancreatic cancer (Supplementary Fig. 2A–D). Meanwhile, bioinformatic analysis indicated that ATG7 expression was upregulated and negative with the prognosis in the pancreatic cancer (Supplementary Fig. 2E–H). miR-766-5p expression was decreased in circATG7-overexpressing PANC-1 cells and increased in circATG7-knockdown cells (Fig. 5C). We found miR-766-5p overexpression reduced the mRNA levels of ATG7 in PC cells, while miR-766-5p inhibition increased the mRNA level (Fig. 5D). Moreover, we found that miR-766-5p carried binding sites for both circATG7 and ATG7 (Fig. 5E). miR-766-5p decreased luciferase activity in PC cells with wild-type circATG7 and ATG7, but not in those with mutant circATG7 and ATG7 (Fig. 5F). We found that both circATG7 and miR-766-5p were significantly enriched in biotin-labeled miR-766-5p probes compared to control probes (Fig. 5G). Interestingly, evaluation of circATG7, miR-7665p, and ATG7 expression using RT-qPCR revealed strong correlations (Fig. 5H). In conclusion, these results indicate that cytoplasmic circATG7 increases the mRNA level of ATG7 by sponging miR-766-5p.

### Nuclear circATG7 increased the stability of ATG7 mRNA by recruiting HUR

**Fig. 6:**
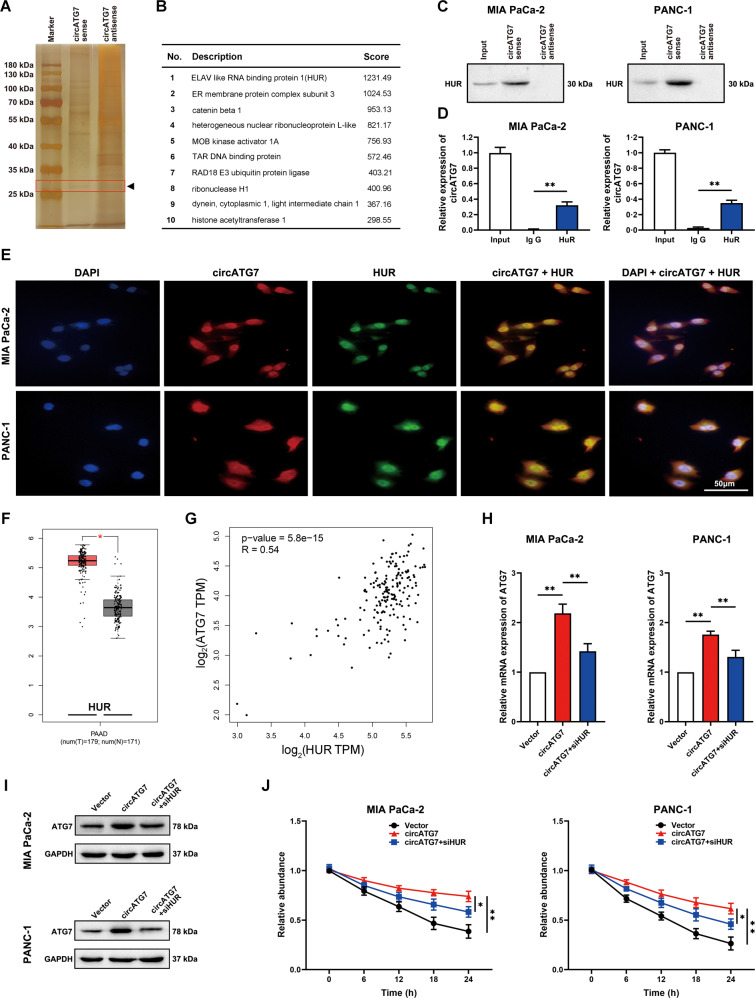
Nuclear circATG7 increased the stability of ATG7 mRNA by recruiting HUR. **A**, **B** RNA pull-down demonstrated that HUR significantly binds with circATG7. **C** Western blotting was used to confirm the binding of circATG7 with HUR. **D** HUR antibodies were used to evaluate circATG7 enrichment. **E** Confocal experiments were used to observe the co-localization of circATG7 and HUR in the cell nucleus. **F** Expression of HUR in PC tissues and adjacent normal tissues based on the data from TCGA. **G** The relationship between circATG7 and HUR was determined according to the data from TCGA. **H**, **I** ATG7 expression was detected using RT-qPCR and western blotting in PC tissues with circATG7 overexpression plus HUR inhibition. **J** The stability of ATG7 mRNA was evaluated in PC cells with circATG7 overexpression plus HUR inhibition. *P < 0.05; **P < 0.01.

RNA pull-down analysis revealed that several proteins, including HUR, ER member protein complex subunit 3, catenin beta 1, heterogeneous nuclear ribonucleoprotein L-like, MOB kinase activator 1 A, TAR DNA binding protein, RAD18 E3 ubiquitin protein ligase, ribonuclease H1, light intermediate chain 1, and histone acetyltransferase 1 could bind to circATG7. Among them, HUR had the highest binding score (Fig. 6A, B). Immunoprecipitation with an HUR-antibody revealed that circATG7 was significantly enriched with HUR protein (Fig. 6C). Similarly, HUR antibodies significantly enriched circATG7 (Fig. 6D). CircATG7 and HUR were mostly co-localized in the cell nucleus (Fig. 6E). Moreover, we found that HUR was significantly increased in PC tissues compared with adjacent tissues (Fig. 6F), and positively associated with ATG7 expression in PC tissues (Fig. 6G). Knockdown of HUR in circATG7-overexpressing PC cells significantly reversed the accelerative effects of circATG7 on the protein and mRNA levels of ATG7 (Fig. 6H, I). However, in the PC cells transfected with HUR siRNA and miR-766-5p inhibitor, ATG7 mRNA expression were almost no difference in circATG7 overexpression or inhibition group (Supplementary Fig. 3). Increased circATG7 significantly decreased the rate of ATG7 mRNA degradation, while knockdown of HUR in circATG7-overexpressing cells increased the rate of degradation (Fig. 6J). These results indicate that nuclear circATG7 may increase the stability of ATG7 mRNA by recruiting HUR.

### CircATG7 promoted the proliferation, motility, and autophagy in vitro via miR-766-5p and ATG7

**Fig. 7:**
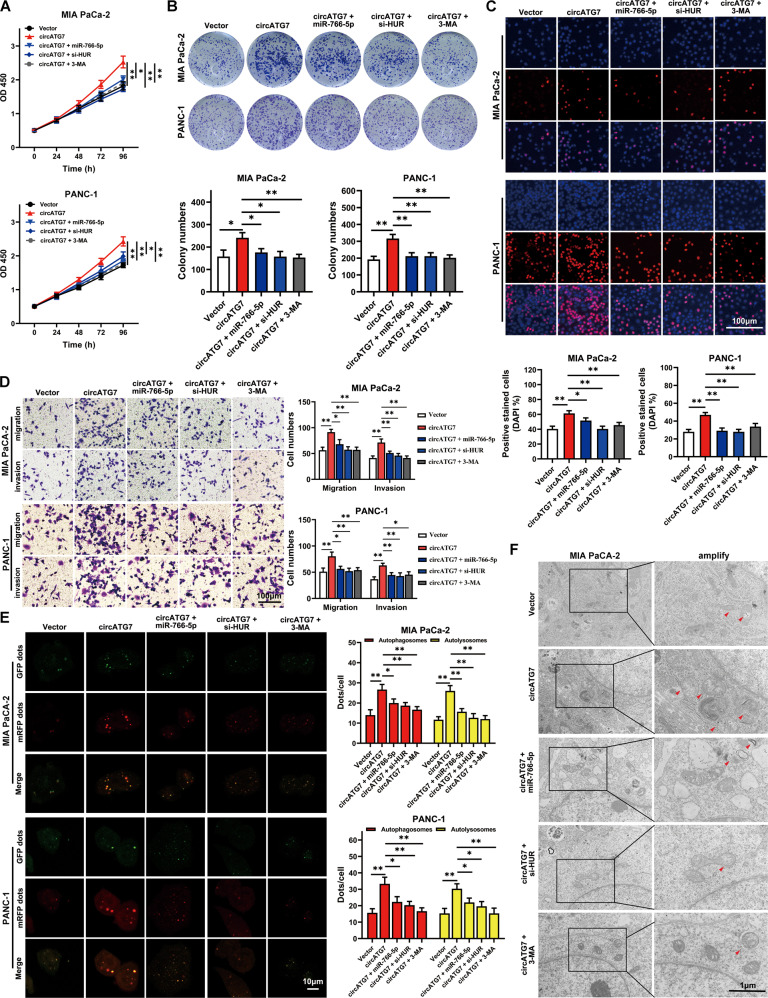
CircATG7 promoted the proliferation, motility, and autophagy in vitro in a miR-766-5p- and ATG7-dependent manner. PC cells were treated with empty vector, circATG7-overexpressing lentivirus, circATG7-overexpressing lentivirus plus miR-766-5p mimic, circATG7-overexpressing lentivirus plus HUR inhibition and circATG7-overexpressing lentivirus plus 3MA. **A** CCK-8 assay was used to determine the rate of PC cell proliferation in each group. **B** EDU assay was performed to detect the rate of EDU positivity in each group. **C** Colony formation assay was used to detect colony formation in each group. **D** Migration and invasion Transwell assays were used to determine the rates of migration and invasion in each group. **E**, **F** Confocal and transmission electron microscopy were used to detect autophagy in each group of PC cells. Red arrows represent autophagosomes. *P < 0.05; **P < 0.01.

CCK8 and EDU assays revealed that miR-766-5p, HUR inhibition, and autophagy inhibitor 3MA(3-Methyladenine) significantly decreased the facilitating effects of circATG7 on PC cell proliferation (Fig. 7A, B). The results of the colony formation assay showed that suppression of miR-766-5p, HUR inhibition and 3MA(3-Methyladenine) in circATG7-overexpressing PC cells reduced colony formation (Fig. 7C). Similarly, Transwell migration and invasion assays revealed that migration and invasion were significantly reduced in PC cells co-transfected with circATG7 lentivirus/miR-766-5p mimic, circATG7 lentivirus/HUR siRNA and circATG7 lentivirus/3MA compared with cells transfected with circATG7 lentivirus (Fig. 7D). Moreover, the levels of autophagy were markedly decreased in PC cells co-transfected with circATG7 lentivirus/miR-766-5p mimic, circATG7 lentivirus/HUR siRNA, and circATG7 lentivirus/3MA compared with cells transfected with circATG7 lentivirus (Fig. 7E, F).

### CircATG7 promote the proliferation, metastasis, and autophagy of PC cells in vivo

**Fig. 8:**
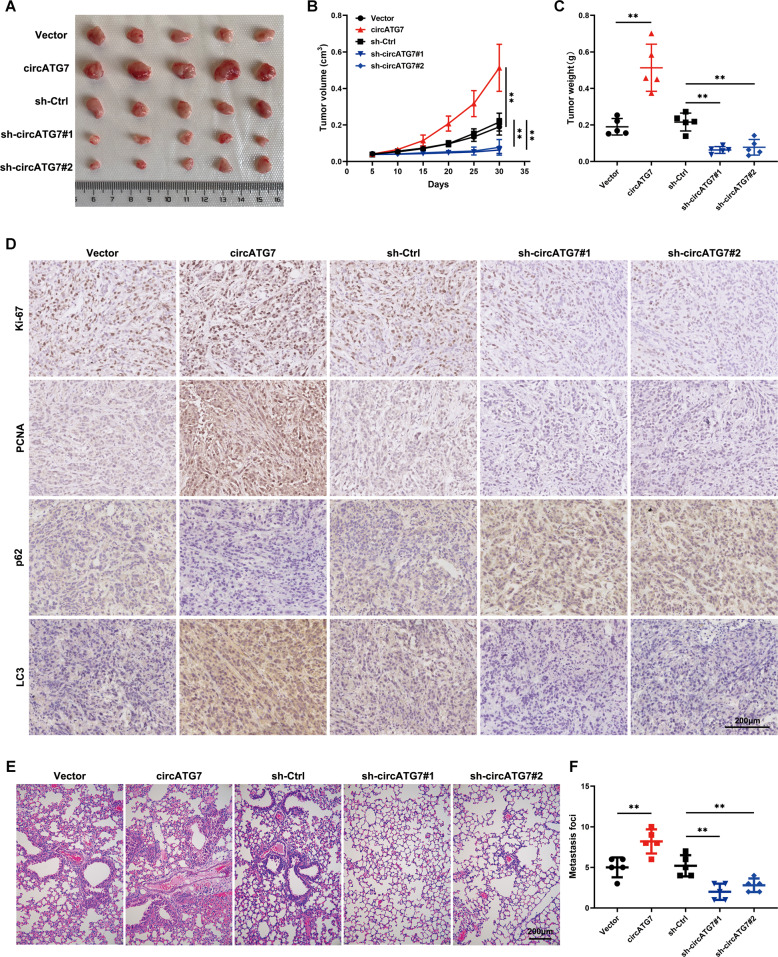
CircATG7 promoted the proliferation, motility, and autophagy of PC cells in vivo. **A**, **B** The rate of proliferation in tissues with circATG7 overexpression and circATG7 knockdown. **C** The weight of tumor tissues with circATG7 overexpression and circATG7 knockdown. **D** The expression of PCNA, KI67, P62, and LC3 was determined in tumor tissues with circATG7 overexpression and circATG7 knockdown using immunohistochemical staining. **E**, **F** A metastatic lung model was used to detect the metastatic ability of PANC-1 cells with circATG7 overexpression and circATG7 knockdown. **P < 0.01.

Experiments with a xenograft tumor model showed that tissues overexpressing circATG7 had a higher growth rate compare with those with normal circATG7 expression, and loss of circATG7 induced a decreased growth rate (Fig. 8A, B). Similarly, tissues overexpressing circATG7 had a higher weight, while those with knocked-down circATG7 had a lower weight (Fig. 8C). Moreover, KI67, PCNA, and LC3 expression was increased in tissues overexpressing circATG7, while P62 expression was decreased. Compared with tissues in the negative control group, those with lower circATG7 expression exhibited lower KI67, PCNA, and LC3 expression, and higher P62 expression (Fig. 8D). Moreover, lung tissues in the circATG7 overexpression group had more metastatic foci, while those in the circATG7 knockdown group had fewer metastatic foci (Fig. 8E).

## Discussion

In recent years, advances in the development of sequencing technology have led to the systematic identification of circRNAs, and their biological functions have been widely studied. Due to the closed-loop structure and lack of 5′ caps and 3′ tails, circRNAs are more stable than their linear parents [25]. Several circRNAs are involved in the progression of PC. In the present study, we found that a novel circRNA, termed circATG7, was increased in PC tissues compared to adjacent normal tissues. Cytoplasmic circATG7 and nuclear circATG7 were found to promote autophagy, proliferation, and metastasis via the miR-766-5p/ATG7 and HUR/ATG7 axes, respectively.

In recent years, circRNAs have been shown to be significantly dysregulated in PC tissues compared with adjacent tissues, and either promote or suppress the progression of PC [26]. Accumulated researches brought forward that the crosstalk between circRNAs and autophagy probably exert essential function involving in the tumor development [27, 28]. Meanwhile, appropriate degree of autophagy can promote the physiological homeostasis of tumor cells, and mediate cell protection by degrading proteins, organelles, or metabolites [29]. Therefore, clarifying the correlation of circRNAs and autophagy seems particularly important to the research about pancreatic cancer. In the study of autophagy-related circRNA, it has been found that there is an interactive regulatory relationship between circRNA and autophagy in non-small cell lung cancer [30], bladder cancer [31], and prostate cancer [32]. However, the regulatory relationship in pancreatic cancer is not clear. ATG7 is a well-characterized mediator of autophagy via recruitment of the ATG5-12 complex which is highly expressed in PC tissues and promotes the progression of PC [33, 34]. In the present study, we found a novel circRNA produced by the ATG7 gene, termed circATG7, which was significantly elevated in PC tissues and cell lines compared to adjacent normal tissues and normal pancreatic epithelial cells. Patients with high circATG7 expression have a poor overall survival rate. Interestingly, circATG7 expression was higher in PC tissues with high LC3 dots, and positively associated with the number of LC3 dots in PC tissues. We found that circATG7 was located in both the cell cytoplasm and nucleus. Moreover, gain and loss experiments revealed that circATG7 overexpression promoted the proliferation, motility, and autophagy in vitro and in vivo, while knockdown of circATG7 induced the opposite effects. These findings suggest that circATG7 may be a novel autophagy-related circRNA and may act as onco-circRNA in PC.

Previous studies have shown that circRNAs regulate biological functions in cells via a parent-dependent mechanism [23]. Therefore, we speculated that circATG7 promotes PC progression by regulating ATG7. Consistent with this, we found that inhibition of ATG7 significantly reversed the accelerative effects of circATG7 on PC cell proliferation, motility, and autophagy. As shown in previous studies, circRNAs can act as ceRNAs to sponge miRNAs and regulate gene transcription; interact with RNA binding proteins and increase the stability of target mRNAs; or be directly translated into proteins [35]. The ceRNA mechanism is common in the cell cytoplasm, while nuclear-localized circRNAs interact with RNA binding proteins. A cytoplasmic circRNA, circ-solute carrier family 8 member A1, sponges miR-130b/miR-494 and suppresses the progression of bladder cancer [36]. The nuclear circ-DLC1 Rho GTPase-activating protein interacts with HUR, and decreases the stability of MMP1 mRNA, resulting in suppressed proliferation [37]. In the present study, circATG7 was found to be located in both the cell nucleus and cytoplasm; therefore, we consider that circATG7 regulates its parent via two mechanisms. The results of bioinformatics analyses, luciferase activity assays, and molecular biological experiments, revealed that miR-766-5p is a key miRNA linked to cytoplasmic circATG7 and ATG7 mRNA. CircATG7 sponges miR-766-5p and increases the expression of ATG7 in PC cells Next, we determined the nuclear mechanism of circATG7 in PC. Through RNA-pull down and confocal microscopy, we found that circATG7 binds to and co-localizes with HUR in the cell nucleus. Knockdown of HUR significantly decreased the stability of ATG7 Moreover, we found that both miR-766-5p and HUR inhibition reversed the accelerative effects of circATG7 on PC cell proliferation, motility, and autophagy.

Taken together, our results indicate that the autophagy-related circATG7 acts as an onco-circRNA in PC. It promotes proliferation, metastasis, and autophagy via the miR-766-5p/ATG7 and HUR/ATG7 axis. Targeting circATG7 may be a potential therapeutic strategy for patients with PC.

## Supplementary information

Reproducibility checklist

Supplement Figure 1

Supplement Figure 2

Supplement Figure 3

Supplement figure legends

The full and uncropped western blots

## Data Availability

All data needed to evaluate the conclusions in the paper are present in the paper. Additional data related to this paper may be requested from the corresponding author.
