# Supplementary figures and images for: Autophagy-associated circRNA circATG7 facilitates autophagy and promotes pancreatic cancer progression
Source: Cell Death Dis. 2022 Mar 14;13(3):233. doi: 10.1038/s41419-022-04677-0 (PMC8921308; doi:10.1038/s41419-022-04677-0)

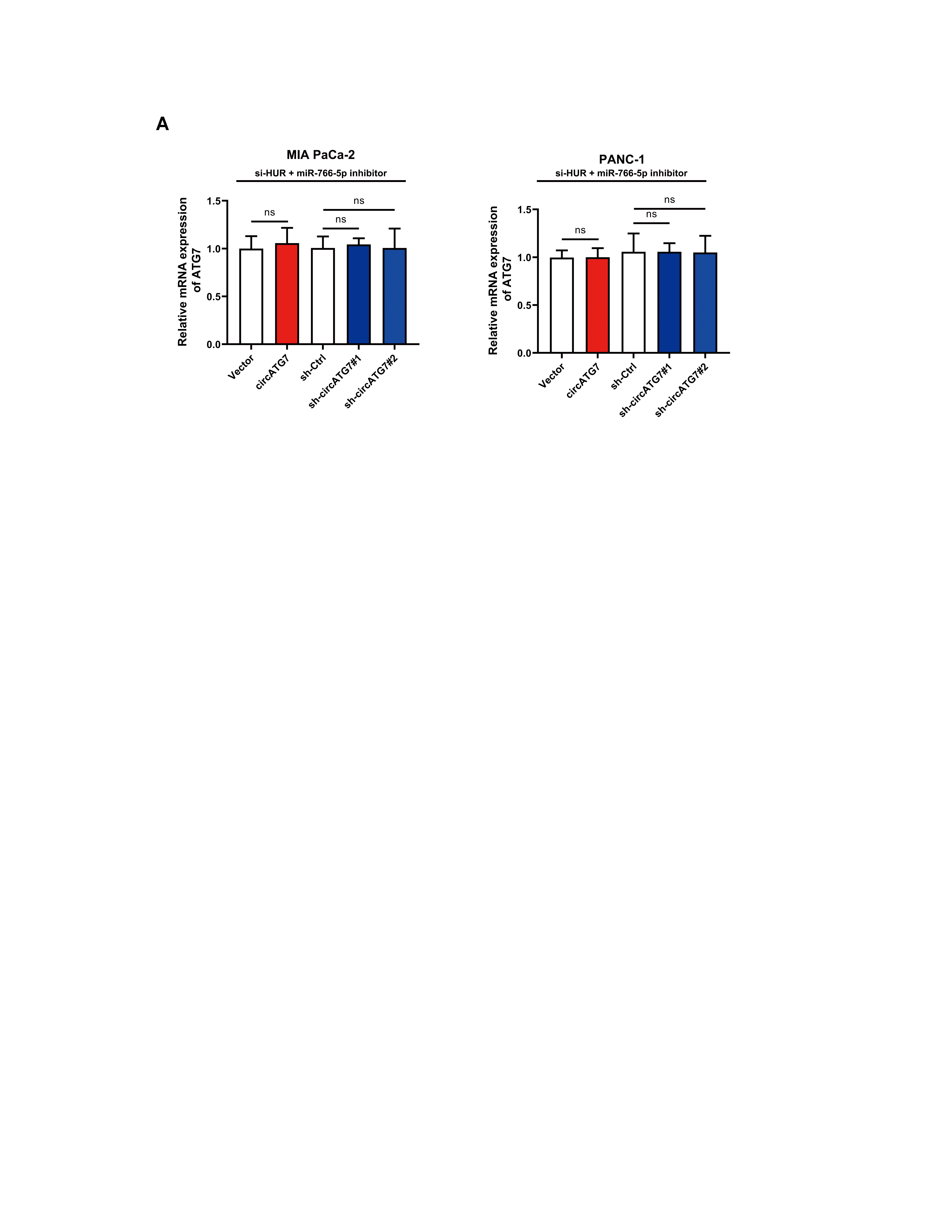

Supplement: Supplementary file 4 — Supplement Figure 3 [file 41419_2022_4677_MOESM4_ESM.tif]

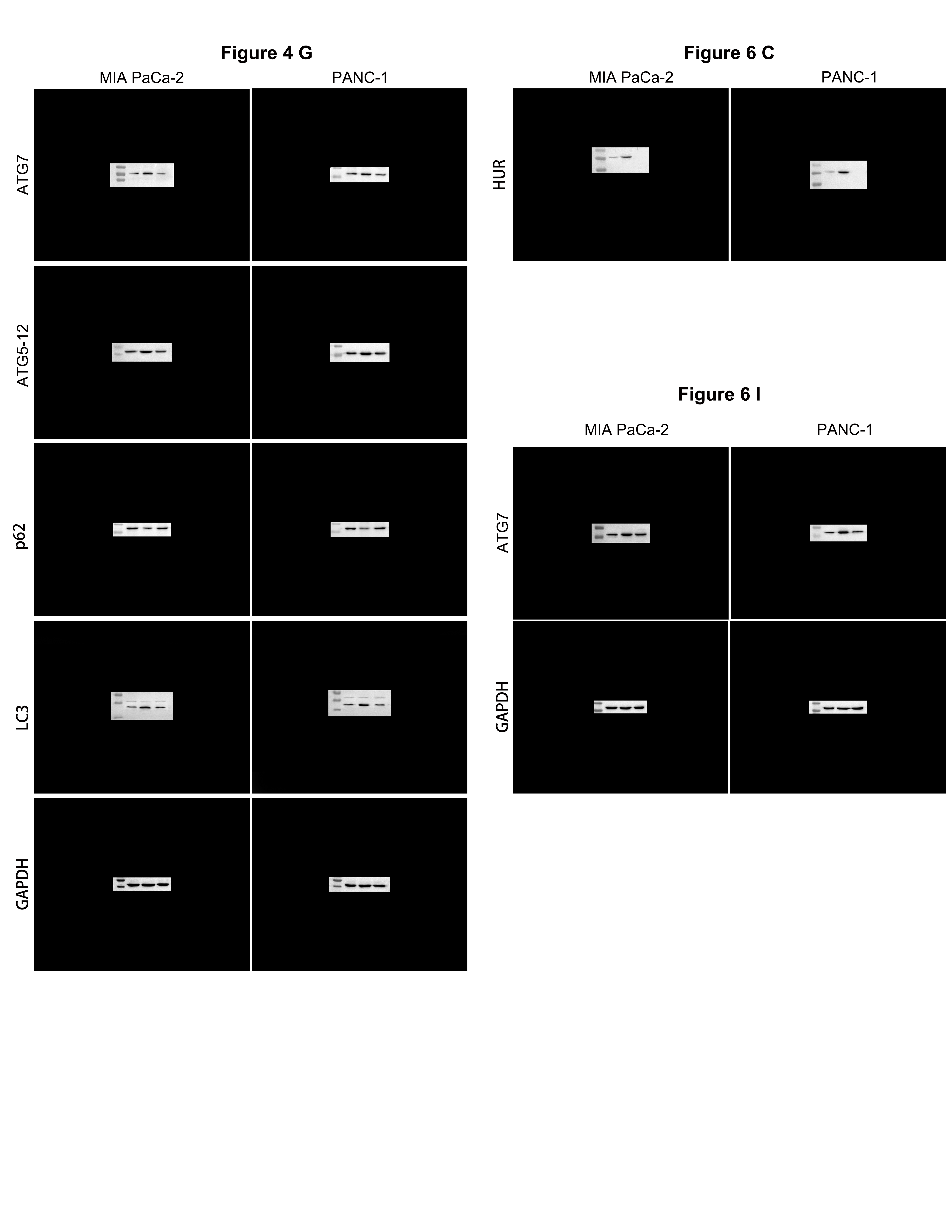

Supplement: Supplementary file 6 — The full and uncropped western blots [file 41419_2022_4677_MOESM6_ESM.tif]
