# Supplementary material for: Autophagy-associated circRNA circATG7 facilitates autophagy and promotes pancreatic cancer progression
Source: Cell Death Dis. 2022 Mar 14;13(3):233. doi: 10.1038/s41419-022-04677-0 (PMC8921308; doi:10.1038/s41419-022-04677-0)
Supplement: Supplementary file 5 — Supplement figure legends [file 41419_2022_4677_MOESM5_ESM.docx]

**Supplement Figure 1.**

**A** The standard PRISMA flow of autophagy-associated circRNA in PC.  **B** Representative cases of PC tissues with LC3-dotsless and LC3-dotsmore are shown. **C** Kaplan–Meier survival analysis showing the overall survival for PC patients with LC3 dots^less^ and LC3 dots^more^.

**Supplement Figure 2.**

**A** Expression of miR-766-5p in PC tissues and adjacent normal tissues based on the data from TCGA. **B**  Expression of miR-766-5p in PC tissues and adjacent normal tissues based on the data from ICGC. **C** Kaplan–Meier survival analysis showing the overall survival for PC patients with high and low expression of miR-766-5p based on the data from TCGA. **D** Kaplan–Meier survival analysis showing the overall survival for PC patients with high and low expression of miR-766-5p based on the data from ICGC. **E** Expression of ATG7 in PC tissues and adjacent normal tissues based on the data from TCGA. **F** Expression of ATG7 in PC tissues and adjacent normal tissues based on the data from ICGC. **G**  Kaplan–Meier survival analysis showing the overall survival for PC patients with high and low expression of ATG7 based on the data from TCGA. **H** Kaplan–Meier survival analysis showing the overall survival for PC patients with high and low expression of ATG7 based on the data from ICGC.

**Supplement Figure 3.** In the PC cells transfected with HUR siRNA and miR-766-5p inhibitor, ATG7 expression was detected using RT-qPCR in circATG7 overexpression or inhibition group.
